# Tableaux of New Orleans

**Published:** 1852-07

**Authors:** 


### BIBLIOGRAPHICAL NOTICES.

---

Art. V.—*Tableaux of New Orleans.* By Bennet Dowler, M. D.

This pamplet gives a great deal of valuable information, geographical, commercial, geological, and sanitary, respecting one of the most important cities in our country. It is written with all the learning and laborious industry for which its author is distinguished. Under the head of "*The Necropolis of New Orleans,*" he has the following:

In the year 1540 I ascertained, by careful inspection, the ages of a great number of persons buried in the cemeteries of New Orleans, copied from monumental inscriptions, taken at random, that is, without picking or selection, in series of thirty each. The numerical results of this inquiry, in the absence of more authentic registries of births, deaths and ages, may be valuable in determining approximatively the duration of life, and the sanitary history of the climate of New Orleans. The necrological inscriptions in the different cemeteries of this city are not only illustrative of its vital history and sanitary perturbations, but are also, to a considerable extent, characteristic and peculiar.

The ideal of vital statistics, as a method, presents sundry conceptions as absolutely true, in advance of empirical processes, and their actual results in relation to both the statics and dynamics of population. But how clear soever the theoretical principle may be, its useful application is alone to be found in the experimental.

For scientific purposes, the facts of vital research and reasoning must be numerous, continuous, and must extend through long cycles of time. The most accurate record, comprehending the history of every individual in existence for one year, or one dozen of years, would utterly fail to solve some of the most important vital problems, which anterior to experience and its results, are known to be solvable, and within the legitimate range of empirical possibility. Synthetical reasoning determines, in advance of experience, that the mean duration of life in a country can be inferred from a record of the ages of all the natives dying within its limits. The record of one entire generation would not only show the exact duration of the mean life for that period, but in connection with a sufficient number of similar previous records, would indicate, with slight and unimportant variations, the mean duration and expectation of life for succeeding generations. In the science of population, however, a generation commencing at a given era, would not be complete as a vital cycle until the last individual then living shall die. For example, an individual born in 1851, might not die until 2020, provided he should live as long as Henry Jenkins did—that is, 169 years. A registry of an entire generation will have been completed at the death of the oldest individual. The sum of all the ages of a given generation, divided by the whole number of individuals, will give the mean age.

Without any regard to the actual census, suppose the fixed creole and acclimated population of New Orleans to be 100,000, and that immigration and emigration be wholly arrested;—suppose four children to be born and four persons to die annnually for every one hundred inhabitants; then the population will be stationary, without increase or decrease, and how much soever individual ages may differ, the average life will be twenty-five years. But in case only two die in the hundred, the births undiminished, the population will increase annually two in the hundred, or for the city two thousand; the whole number of births will be four thousand, and the whole number of deaths two thousand; then the average life will be double—will be fifty instead of twenty five years. Suppose that during the year 100,000 persons come temporarily into the city, and lose two in every hundred, all the survivors returning home, then the deaths and births will be equal, the average life will seem to be reduced to twenty-five, and the increase neutralised; all of these conclusions would be necessarily false, for want of correct data, comprehending all the essential elements entering into the numerical process, static and dynamic.

Although four births and four deaths annually for every one hundred persons would cause the population to be stationary, and the mean life to be twenty-five, yet, with respect to the latter, a single year might give a result altogether different; and so of a very limited number of years; thus, one dying aged one hundred, another fifty, a third twenty, and a fourth ten, the average might be double that which has been named. But in a period sufficiently prolonged, such excesses would be reduced or equalized. In tossing a dollar in the air the same side might fall uppermost for several consecutive times; but if the operation be continued sufficiently long, this marked inequality disappears forever. The same principle prevails in vital statistics, as well as in numerical medicine.

There are, indeed, a great many elementary questions which it is desirable or necessary to consider, in order to arrive at satisfactory conclusians concerning the vital condition of New Orleans.

What is the annual mean number of the resident creole and acclimated population? What is the annual mean number of strangers, and time of their temporary residence? What is the mean number of births, deaths and ages of each class, not to mention internal and external relations, topograthical, ethnographical, physioiogical, sanitary, industrial, pecuniary, educational, moral, religious, social, civil, in the white, black, red, and mixed races?

By the general custom of mankind—one not only in accordance with good taste, but with sanitary requirements—the dead are consigned to the ground—"earth to earih;" but in New Orleans a different method of sepulture prevails. In most of the cemeteries, interment in the ground is wholly interdicted, elevated vaults and tombs only being used. The necessity of this method of entombment, for all who can afford the expense, is easily explained by referring to the topography of the city. A grave in any of the cemeteries is lowor than the adjacent swamps, and from ten to fifteen feet lower than the level of the river, so that it fills speedily with water, requiring to be bailed out before it is fit to receive the coffin, while during heavy rains it is subject to complete inundation. The great Bayou Cemetery is sometimes so completely inundated that inhumation becomes impossible until after the subsidence of the water; the dead bodies accumulating in the meanwhile. I have watched the bailing out of the grave, the floating of the coffin, and have heard the friends of the deceased deplore this mode of interment. A young Irish woman, on seeing her husband's coffin lowered into a grave of welling water, exclaimed repeatedly; "Oh, Mike, it is a dear burying to you to be buried at the Bayou! Oh that you should come to this!" It is this feeling that has built the different cemeteries which constitute the great necropolis of New Orleans. Interest, to say nothing of the vanity of friends, requires inscriptions to identify a vault, which is private property, purchased under a written title or conveyance. Hence, these monumental inscriptions, from their constancy, accuracy and number, afford data which, in the absence of exact registries, are probably more trustworthy and valuable than can be found in any other existing necropolis. These necrological monuments will augment from generation to generation, and must hereafter prove more useful to the vital historian than the pyramids of Egypt, or the countless millions so carefully embalmed and deposited in the catacombs of that country, forty centuries ago. The ethnologist might even now commence his lesson among the tombs. The Caucasian is separated from the negro race. In some cemeteries the Irish, in some the German, in some the Anglo-American, in some the French type predominates.

The monumental evidence to be offered in this tableau, in relation to the salubrity of the city and the length of life, compared with other places, is doubtlessly imperfect. The principal objection to which it is liable, appears to be this; namely, very young children may not have had inscriptions on their vaults, as constantly as adults; though this hypothesis may be incorrect. But admitting that it is true, this source of error is neutralized, it may be supposed, by an undeniable fact, that in all the cemeteries, even those which reflect the creole life most truly, as the Catholic, strangers, victims to the climate, who "liveth not half their days," are buried, and being counted, tend to shorten the average life probably as much as the supposed omission of infantile inscriptions tends to enhance it. The evidence, upon the whole, if not demonstrative possesses probability, and is offered for what it is worth, in the absence of more exact data.

In the following enumerations, fractional parts of a year are reckoned as one year when they exceed six months, or fall short of eighteen months, and so of all fractions in more advanced ages. In all cases it was deemed necessary, in recording a series of ages, not to reject any because they were short, nor to seek any because they were long. Thus, on one occasion, having completed the series for the time and the place, I came immediately to an inscription upon a well known negress, aged 107 years and 5 months, born in 1732, died in 1839, but the rule adopted excluded this, as well as other similar cases. In Lafayette cemetery, as the sexton informed me, there is a negress slave buried aged 110. A similar age was found in the Catholic cemetery, after having finished the series. But all these were omitted.

The old Catholic cemetery, (No. 1, Basin street,) in which nearly all the inscriptions are French, 13 only were distributed among all other languages, gave the following results, after having made 136 observations:

| | | | | | | | | | |
|---|---|---|---|---|---|---|---|---|---|
| The first series of | | 30 | observations gave an aggregate of | | | | | 1474 | years. |
| The second | " " | 30 | " | " | " | " | " | 1512 | " |
| The third | " " | 30 | " | " | " | " | " | 1381 | " |
| The fourth | " " | 30 | " | " | " | " | " | 1313 | " |
| The fifth | " " | 16 | " | " | " | " | " | 852 | " |

Total observations, 136. Total ages 6537:

mean life 48 years and a fraction: more than 21 years over the mean of the Hebrew cemetery—20¼ over that of the Bayou; 17¼ over that of the Protestant;—27⅓ over that of Lafayette city;—12 over that of all France;—nearly 20 over that of the department of the Seine, (Paris)—and about 22 years beyond the mean of the old Protestant cemetery immediately adjacent. The following table shows the mean age, with the three oldest persons in each series, in this cemetery:

| *Series.* | *Mean Age.* | *Three oldest in each Series.* | | | *Mean Age of Three Oldest.* |
|---|---|---|---|---|---|
| 1st. series | 49.01 | 81 | 80 | 76 | 79 |
| 2nd. " | 50.56 | 76 | 76 | 74 | 75.33 |
| 3rd. " | 46.03 | 85 | 80 | 78 | 81 |
| 4th. " | 43.76 | 85 | 81 | 72 | 79.33 |
| 5th. " | 53.25 | 92 | 90 | 90 | 90.66 |

Although the place of nativity is not always mentioned in these inscriptions, yet out of Louisiana the United States furnished but 1, and Ireland but 1, France 19, and Spain, Genoa and St. Domingo, each 4. The prevailing type, in this cemetery, is doubtlessly the creole French.

The old Protestant cemetery, (adjoining the Catholic cemetery on Basin street) long abandoned as a place of burial, gave for 30 inscriptions an aggregate of 797 years, and a mean life of nearly 26½ years—the three oldest, 62, 60, 47.

The new, and by far the most extensive of the Catholic cemeteries, is that in the rear of the former, consisting of four squares, between Robertson and Claiborne streets, the southern portion of which is for the colored race. In this cemetery, especially in its northern portion, French inscriptions preponderate. The white race, in 80 observations, afforded the following results: The first 30 gave an aggregate of 1296, and a mean of 42.2 years—the 3 oldest 89, 77, and 74; the second 30 gave a total of 1415; a mean of 47.16; the 3 oldest 80, 75, 72; the residue 20 observations gave a total of 997; a mean of 49.85 years; the 3 oldest 83, 80, and 75.

The aggregate of these 80 observations amount to 3678 years, giving a mean age of nearly 46. (After counting these 80, one was found aged 110, though I could not count it consistently with my plan, which rejected the principle of selection.) In the middle division of this cemetery, 30 inscriptions gave an average life of nearly 47½ years.

By uniting these divisions of the Catholic cemetery No. 2 with that on Basin street, the observations will amount to 396—the aggregate 18,607 years, and the mean life of the whole, both of the whites and blacks, will be very nearly 47 years.

Of these 396 inscriptions, 49 were over 70;—13 were over 80;—5 over 90.

The black race in this cemetery, buried in a style of magnificence nearly equal to the white, has usually French inscriptions, indicating as the principal places of nativity, Louisiana, St. Domingo, Cuba, Jamaica, and Africa, and gave, in 150 observations, the results which the following table expresses with the utmost brevity: There may be some error in the third series—a discrepancy there seems to be, inasmuch as this series gives a comparatively diminished total and mean life.

| *Series Thirty Obs. Each.* | *Aggregate Ages Of Each Series.* | *Mean Ages of Each Series.* | *Three Oldest In Each Series.* | | |
|---|---|---|---|---|---|
| 1st. Series, | 1594 | 53.13 | 100 | 85 | 80 |
| 2nd. " | 1364 | 45.46 | 64 | 80 | 75 |
| 3rd. " | 1102 | 37.4 | 95 | 82 | 70 |
| 4th. " | 1318 | 43.93 | 100 | 83 | 79 |
| 5th. " | 1585 | 52.0 | 100 | 92 | 90 |
| Total ages of 150, | 6,969 | | | | |
| Mean age of 150 persons, - - | 43.43 years. | | | | |

The united ages of the fifteen oldest persons in this enumeration amount to 1,398 years, affording an average life far beyond "threescore and ten," (the limit indicated by the royal poet of the Hebrews,) namely, 86½ years, with two centenarians for every hundred; or as many of that age as France affords in about a half million. Probably the entire number of vaults and tombs in the African cemetery does not exceed two thousand, nor the dead bodies exceed three thousand. Now, on the supposition that by some strange and incredible chance, the one hundred and fifty inscriptions I took note of, actually exhausted the whole number of centenarians, (which I know was not true,) still the colored centenarians, transcend French centenarians, two hundred and fifty times.

It will be seen that the black race affords, by these tables, 1 in 50, aged 100 years; and if we add 11 years to the lives of the remaining two oldest in the 150 enumerated, the result will be, five centenarians; or 1 in 30; or 8,333 times more than the ratio for all France; or 2,100 more than that of England, by the census of 1841; or if we take the official account of the deaths in France for the 15 years ending on the first of January, 1852, it will be found that 150 inscriptions give for the black race in New Orleans, nearly one fifth as many centenarians as 11,793,-289, or nearly twelve millions, of deaths among the French. But, by an exact calculation, the French bills of mortality, as above mentioned, give one aged one hundred in every 471,731; the black one in fifty.

Each of the remaining cemeteries of New Orleans, as they contain a greater proportion of strangers, will be found to offer a rapid decrement in the mean life. The new and extensive Protestant cemetery of the Second Municipality gave, in the first 30 observations, as the three oldest, 73, 43, 40; the second 30 gave, for the three oldest, 78, 69, 66. From 110 observations, a mean life was obtained of $30\frac{3}{4}$ years. The Hebrew cemetery gave, as the three oldest, 74, 63, 62, and an average of 27 years.

The Bayou cemetery, or Potter's Field, not having monumental inscriptions, with few exceptions, proved an unsuitable field for necrological researches. From the rude and frail memorials of the dead, I obtained thirty-five ages; the oldest three were, 55, 52, 45—the mean life of the whole, $27\frac{3}{4}$ years—a mean nearly twenty years less than that of the old Catholic, and the African cemeteries.

The city of Lafayette, separated from New Orleans by a street only, abounds with German immigrants, who, with the Irish, are in both cities the principal victims of yellow fever. The Lafayette cemetery is more favorable for inhumation in the ground than the New Orleans cemeteries; accordingly, this mode of sepulture is more common in the former. Among 30 ages taken from the vaults of that cemetery, 39 was the oldest, and the mean of the whole was only $20\frac{3}{4}$ years, which is the minimum of all the cemeteries, being 26 years less than that of the black race in the Catholic cemetery, and nearly two and a half times less than that on Basin street.

The Catholic cemeteries are supposed to reflect the creole life more accurately than the other cemeteries, which are newer, and have been filled with immigrants. The mean life, as deduced from monumental evidence, though not identical with that deduced from the recent mortality of the city, by the Board, is confirmed by the latter; that is to say, the Catholic cemeteries take precedence of the Protestant, and the Protestant of the Potter's Field. Any one acquainted with the different classes of the population would have anticipated these results.

The following is interesting, on the "*Sanitary History of New Orleans:*"

There is not, probably, any considerable city in North or in South America, including the West Indies, whose sanitary history is so favorable as that of New Orleans, until near the close of the last century. Not long after the foundation of the city, the Parisian press teemed with works on Louisiana, its climate, topography, botany, zoology, and its native Indians. Some of these works were of an official character, having originated in scientific expeditions ordered by the French Government. Charlevoix's work, [3 vols. 4 to.] describes the city 4 years after its foundation in 1718,—animadverts upon its topography, estimates its population in January, 1722, at 200, who lived in huts upon the river's bank, the regular houses not having been as yet constructed. Laval's work on Louisiana was published in 1728. In neither of these works is the salubrity of the climate questioned. Dr. Pratz, in his history, [3 vols. 1758,] maintains that life in Louisiana was not only pleasant but long. La Harpe, the agent of the French Government, sent to explore the boundaries, climate, &c., of Louisiana, arrived at New Orleans in the fall of 1718. In 1724, after having spent five years in Louisiana, chiefly in New Orleans, he returned to France. His journal is minute, but contains no account of fevers, or other epidemics in the colony. He estimates the inhabitants of the city, including the troops, at sixteen hundred—the climate as being temperate—the air as salubrious; [355, 356.] The people, he continues, were entirely exempt from the epidemics which desolated other parts of North America. New comers, for the most part, were attacked with a slight fever [*une fivre lente*] which caused debility, but never death; [on ne voit pas de personnes en mourir.] The province, which had a black population of 1,000, was everywhere salubrious, particularly along the sea shore.

Lozieres, in his second voyage to Louisiana, 1794 to 1798, [2 vols., Paris, 1803,] asserts the salubrity of New Orleans, and seems much puzzled to explain it. He concludes, however, that the cause must be sought in the universal use of the Mississippi water; [T. i., 313.]

In a work published in Paris in 1802, edited by Duvallon, founded on a three year's actual residence, Louisiana is described as exempt from epidemic diseases, its fevers were mild, and rarely dangerous; the rate of mortality was small; whites and blacks of both sexes lived long, and were still fresh and vigorous at the age of sixty. The writer gives a flattering account of the physical conformation, fine complexion, and rare beauty of the *dames* creoles. The sanitary condition of the city he maintains as being good, with the exception of the yellow fever. He says that this malady was not proper to the place, and had not been known until within the six or seven years preceding 1802, a period during which it had prevailed nearly every summer, and was attributable to the commercial intercourse with the North Americans, and to the increased heat of the climate incidental to the clearing away the forest, and the exposure of the soil to the sun's rays.

This author asserts that from the end of October to the beginning of July, there is in both town and country but little sickness, and that death but rarely occurs. During the hot season of three years preceding 1803, the mercury arose no higher than from 24° to 26°.; [Reau,] nor did it descend in the winters lower than two degrees below the freezing point.

Robin, in his travels in Louisiana from 1802 to 1806, [3 vols., Paris, 1817,] avers that the country has but few chronic diseases, diarrhea being the most common and fatal. He mentions sore throat as sometimes prevailing among children; also, infantile tetanus, [le malde de machoire,] together with yellow fever, which a few years previously had appeared for the first time in the city, and which he identifies by Father Labat's description, enlarging on its pathology as a disease of the blood, or rather as a rarefaction of that fluid, causing dilatation, tension and compression in persons from the North, whose blood he supposes is too rich and dense. Tropical acclimation, according to him, renders the blood thinner, lighter, and less dilatable.

The celebrated statesman, Count de Vergennes, in a memoir on Louisiana, addressed to his Government during the early days of the American Revolution, says: "I repeat what I have so often said, that Louisiana is, without question, the first country in the world as to mildness of climate, and its happy situation."

The immigrant to New Orleans runs a risk of the yellow fever during the first few, say 3 to 4 years, a great proportion escaping altogether, and a few suffering an attack after a more prolonged residence,—one attack with continuous residence protecting against subsequent attacks. His dangers from epidemic fevers are, therefore, compressed within a few years, while in Northern latitudes, or even beyond the limits of the city, he is, during life, exposed to the risks of epidemic, bilious, remittent, intermittent, congestive, typhus, and typhoid fevers.

The yellow fever is emphatically the strangers' fever; individuals long resident, and natives of the city, being as little liable to take this disease as the vaccinated are to contract small pox.

Independent of emigrants, New Orleans receives within its limits for a few days or months a greater proportion of floating population than any other city, arising from its commercial character, geographical position, easy access by rivers, lakes, and seas, and from its public charity, thus becoming the asylum of foreign paupers passing through or temporarily resident in it. To these may be added sick and destitute boatmen, seamen, ditchers, woodchoppers and raftmen from the valley and swamps of the lower Mississippi, not to name a vast many poor consumptives seeking the benefit of the climate. Besides, New Orleans has been, and continues to be, the recipient of the broken down soldiers, the *debris* of the armies of Generals Taylor and Scott. The unparalleled California emigration contributes in the same way.

It is a problem unsolved by experience, whether a migration of the natives of these hot countries to the icy circle, would not be more fatal than that of the Northerners towards the torrid zone. Would not northern typhus, with pulmonary diseases, transcend in mortality southern remittent and yellow fever?

But in no case can acclimating diseases be received as the precise standard of salubrity or insalubrity for the entire, much less native population.

The sanitary statistics of New Orleans have been derived, in too many instances, from the books of the Charity Hospital, which is virtually a great foreign institution, located in, but not for New Orleans. Thus, in twenty years, ending in 1850, the admissions of patients into that institution [according to Dr. Fenner's valuable Reports] amounted to 123,917, of which number only 1,293 were Louisianians, that is to say, about one in every hundred. But probably not more than half of these were citizens of New Orleans; that is, one in every two hundred. Admitting, what is generally conceded, that the hospital admissions represent from one-fourth to one-third of the whole number of sick strangers; but taking the latter ratio as a guide, it follows that 371,751 strangers are to be charged to the sick list of the actual citizens of the town.

The report of the Board of Health of New Orleans, for 1849, [one the least flattering to its sanitary character ever made,] shows that in the Catholic cemetery, for four years, ending in 1844, among 442 deaths there were ten aged more than one hundred years! Now, it requires nearly two and a half millions of people in France to produce ten centenarians,* according to the census, and still more according to the official bills of mortality. The entire department of the Seine [Paris] would have only eight centenarians, provided it had *ten millions of inhabitants*,* which is nearly one-third of the population of the entire Republic. New Orleans, according to this ratio, gives, in its necrological record, one in forty-four aged beyond one hundred years; while, by the census of 1840, there is in the United States but one in 6,157 aged one hundred years. In England and Wales there is but one of this age in every 55,555; in France, one in every 250,000. The centenarians in the Catholic cemetery are, therefore, nearly six thousand times greater [in equal numbers] than in France. Even the Potter's Field, according to the Report, in 8,566 gives nine centenarians, or one in every 951, that is, about 26.3 times more than the French Republic.

The Report asserts that "the average age at death in the Northern cities, [doubtless owing in a great measure to the large mortality in infantile life,] is from nineteen years, nine months, to twenty years, three months, and in some of the cemeteries, where destitute foreigners from the crowded parts of the city of Boston are buried, it is reduced to 13.43. * * * * In the city of New Orleans, the average age at death for the last year was 26.69, and for a series of years, the aggregate of all the cemeteries was 22.63." Is an increased average life to be considered a proof of the insalubrity of our climate, espe-

---

*Essai sur la Statistique de la Population Francaise. Par le Comte A. D'Angeville.

cially when that life is twice as great as in some of the cemeteries of Boston? The foreign population of Boston is about equal to the native. Nearly all of the former are Irish. These Irish, in going from their own country to Boston, go to a climate like their own, not to a tropical climate, as in New Orleans—an important consideration.

Now, the Report shows in the Potter's Field, or Bayou Cemetery, [the great Irish necropolis of New Orleans,] that the average life taken for six years, ending in 1846, is nearly twenty-four; that is, nearly one-fifth more than the native and foreign mean life of the most favored Northern cities.

The Report estimates the mortality for 1849 at 9,862, of which number Louisiana and New Orleans furnished less than one in every twelve, and the residue of the United States about one in twenty. Those known to have been foreigners, [3,569,] and those whose places of nativity were not known, that is, 4,985, were probably nearly all foreigners, negroes excepted. The United States, including Louisiana and New Orleans, contributed to the whole number, namely, 9,862, only 1,308. Now, let us give the Report nearly twice as many deaths for the United States, namely, 1,017 more than the number mentioned in the Report, whereby it will appear that foreigners, nevertheless, exceeded Americans between four and five times, or, by the figures of the Report, nearly eight times. Can any other city show a mortality in which *its own share*, including that of the temporary residents of the whole State, is less than one in twelve? Concede to the Report 1,017 deaths of native Americans beyond its own account—admit [what is probably true] that all the centenarians mentioned are natives, and what is the result? The Report gives one aged 130, one aged 110, one aged 105, and twenty aged 100, or twenty-three aged 100 and over; that is, more than one centenarian to every 101, or 2,500 times more than France; or, taking the deaths of Louisiana alone, twenty-three centenarians in twenty-nine deaths, or one centenarian to thirty-three deaths among the natives of the city.

Even the cholera passed by the natives of New Orleans to a very great degree, taking the Report as authority. Thus, of 3,171 deaths which took place from that disease in 1849, the natives of the city and State contributed only

106; or 1 in 30 nearly of the whole. Among 783 deaths from yellow fever, the State and city contributed 2, or 1 in 391.5, and among 640 deaths from all other fevers, but 17, or about 1 in 40.

The mortality of 1849, though great, illustrates the mortuary statistics of New Orleans for other years. Dr. Fenner, in his Medical Reports, shows that in 1849 the total number admitted into the Charity Hospital of New Orleans was 15,558—of these 13 634 were from foreign countries; from countries unknown, 142, probably nearly all foreigners, making 13,776 foreigners; from the different States of the Union 1,782 were admitted, leaving, as he shows, only 147 for all Louisiana; that is, one Louisianian for every 10,397 patients born in other climates.

Dr. Fenner shows that of the whole number admitted into the Charity Hospital in 1849, 17½ per cent. died, making 2,739 deaths. This ratio will give Louisiana about 27 deaths—but several reasons could be adduced rendering it probable that Louisiana did not furnish an average mortality half as great in the 100 as foreigners—say 12 in 147. Here, then, are 12 deaths in 2,727, or if figures be taken without explanation, 147 sick Louisianians furnished, in 1849, no fewer than 2,739 deaths.

Dr. Fenner gives the statistics of the Charity Hospital for twenty years, ending in 1850; admissions, 123,917; of these 1,283 were Louisianians, a little over one in a hundred. Adopting the usual estimate, namely, that the other hospitals and the private cases in the city exceed those of the Charity Hospital in the ratio of two to one, then the last twenty years gave 321,917 cases of sickness, of which 3,879 were from Louisiana—in round numbers, 3,000 to 318,000.

Of 1,800 who died of yellow fever in New Orleans in 1841, the State of Louisiana and its cities contributed but eight, or 1 in 225; the nine most Southern States, including Texas, only twenty-five, or one in seventy-two; and the entire black race only three, or one in six hundred.

The hospitals of Paris are for Frenchmen; the Charity Hospital of New Orleans, the only one in the State of Louisiana, is virtually for foreigners. In Paris one-sixth of the whole population die in the public hospitals.* In

*Dupin Alison.

a population of 70,000, or one in every ten, pass annually through the public hospitals,* while in the Charity Hospital of New Orlhans, the whole State, in twelve years, ending in 1842, supplied, among 59,021 patients, only 556, or 45 annually—that is, one to ,731—a ratio 783 times less than that of Paris. In 1842, among 4,404 patients in the Charity Hospital, Louisiana furnished only 34, not one in ten thousand of the inhabitants, or one thousand times less than Paris. In Dublin, in 1827, more than one in four entered the fever hospitals of that city, namely, 60,000†—a ratio twenty-five thousand times above that of Louisiana.

